# Pre-harvest management is a critical practice for minimizing aflatoxin contamination of maize

**DOI:** 10.1016/j.foodcont.2018.08.032

**Published:** 2019-02

**Authors:** George Mahuku, Henry Sila Nzioki, Charity Mutegi, Fred Kanampiu, Clare Narrod, Dan Makumbi

**Affiliations:** a International Institute of Tropical Agriculture (IITA), P.O. Box 34441, Dar es Salaam, Tanzania; b Kenya Agricultural and Livestock Research Organization (KALRO), P.O. Box 57811-00200, Nairobi, Kenya; c International Institute of Tropical Agriculture (IITA), P.O. Box 30772-00100, Nairobi, Kenya; d University of Maryland, 5201 Paint Branch Parkway, College Park, MD 20742, USA; e International Maize and Wheat Improvement Center (CIMMYT), P.O. Box 1041-00621, Nairobi, Kenya

**Keywords:** Maize, Pre-harvest, Aflatoxins, Exposure, Kenya

## Abstract

Maize, the main dietary staple in Kenya, is one of the crops most susceptible to contamination by aflatoxin. To understand sources of aflatoxin contamination for home grown maize, we collected 789 maize samples from smallholder farmers’ fields in Eastern and South Western, two regions in Kenya representing high and low aflatoxin risk areas, respectively, and determined aflatoxin B_1_ (AFB_1_) using ELISA with specific polyclonal antibodies. AFB_1_ was detected in 274 of the 416 samples from Eastern Kenya at levels between 0.01 and 9091.8 μg kg^−1^ (mean 67.8 μg kg^−1^). In South Western, AFB_1_ was detected in 233 of the 373 samples at levels between 0.98 and 722.2 μg kg^−1^ (mean 22.3 μg kg^−1^). Of the samples containing AFB_1_, 153 (55.8%) from Eastern and 102 (43.8%) from South Western exceeded the maximum allowable limit of AFB_1_ (5 μg kg^−1^) in maize for human consumption in Kenya. The probable daily intake (PDI) of AFB_1_ in Eastern Kenya ranged from 0.07 to 60612 ng kg^−1^ bw day^−1^ (mean 451.8 ng kg^−1^ bw day^−1^), while for South Western, PDI ranged from 6.53 to 4814.7 ng kg^−1^ bw day^−1^ (mean 148.4 ng kg^−1^ bw day^−1^). The average PDI for both regions exceeded the estimated provisional maximum tolerable daily intake of AFB_1_, which is a health concern for the population in these regions. These results revealed significant levels of preharvest aflatoxin contamination of maize in both regions. Prevention of preharvest infection of maize by toxigenic *A. flavus* strains should be a critical focal point to prevent aflatoxin contamination and exposure.

## Highlights

• Aflatoxin in pre-harvest maize was evaluated in high- and low-risk regions of Kenya.
• More than 60% of samples from each region had detectable AFB_1_, up to 9091 μg kg^−1^
• Average probable daily intake for both regions exceeded the PMTDI for aflatoxins.
• Consumption of homegrown maize constitutes a health risk for the population.
• Pre-harvest is a critical step to focus aflatoxin intervention strategies.

## 1 Introduction

Aflatoxins are secondary metabolites produced by *Aspergillus flavus* and *A. parasiticus* fungi that are ubiquitous in many tropical soils where maize (*Zea mays L.*) is grown. Aflatoxins are a serious problem affecting short and long-term health of humans and animals, trade and export markets of maize based products (Misihairabgwi, Ezekiel, Sulyok, Shephard, & Krska, 2017). When consumed in low dosages over prolonged periods, aflatoxins may cause liver cancer, suppress immune systems, increase the incidence and severity of infectious diseases, lead to poor nutrient absorption, retarded child growth and development by contributing to malnutrition (Kensler, Roebuck, Wogan, & Groopman, 2011; Williams et al., 2004). Chronic exposure is a major risk factor for hepatotoxic carcinoma, particularly in areas where hepatitis B virus infection is endemic (Kensler et al., 2011; Wu & Santella, 2012). Ingestion of higher doses of aflatoxin can result in acute aflatoxicosis, which manifests as hepatotoxicity, or in severe cases, fulminant liver failure and death (Kamei & Watanabe, 2005; Lewis et al., 2005).

Maize, the main dietary staple in Kenya, is one of the crops most susceptible to infection by *A. flavus* and contamination by aflatoxin. Contamination of maize grain with aflatoxin has been a major issue in Kenya, where average per capita consumption is 400 g of maize/day (Lewis et al., 2005). More than 75% of maize in Kenya is produced by smallholder farmers and mostly for their own consumption, and the surplus is informally traded. Under this scenario, aflatoxin contamination of home-grown maize presents a significant threat to the health of rural and urban consumers, who are dependent on maize as their staple.

Kenya has witnessed periodic incidences of acute aflatoxin poisoning dating back to 1981 as a result of consumption of aflatoxin contaminated maize (Ngindu et al., 1982). Multiple aflatoxicosis outbreaks have been documented since 2004, resulting in nearly 500 acute illnesses and 200 deaths (Azziz-Baumgartner et al., 2005; Lewis et al., 2005; Kang’ethe et al., 2017). Most reported aflatoxicosis outbreaks have occurred among people living in rural subsistence farming communities in Kenya's Eastern province and were usually associated with consuming homegrown maize (Azziz-Baumgartner et al., 2005; Daniel et al., 2011). Thus, it is unknown whether aflatoxicosis outbreaks and aflatoxin exposure are truly limited to the Eastern province.

Despite the health and economic importance of aflatoxins in Kenya, little has been done to document the incidence and prevalence of aflatoxin contamination of maize during pre-harvest phase when the crop is still in the field. This information is important to better target control strategies to minimize contamination of maize by *A. flavus* and subsequent aflatoxin contamination, and thus, contribute to food security and safety for the rural and urban poor who are dependent on maize. This study was conducted to document the levels of pre-harvest aflatoxin contamination of physiologically mature maize collected from farmers’ fields. To better understand the extent of the aflatoxin problem beyond Eastern Kenya, the study targeted a high maize consumption region of South Western where aflatoxin poisoning has not previously been reported, and compared the results to the high-risk region of Eastern Kenya with numerous reports of aflatoxin poisoning (Azziz-Baumgartner et al., 2005; Daniel et al., 2011; Lewis et al., 2005). This information is critical for determining the total burden of disease attributed to aflatoxin exposure and for targeting public health interventions. In addition, this information is also essential for identifying whether pre-harvest aflatoxin contamination is a critical point along the maize value chain that could be targeted to prevent future outbreaks of aflatoxin poisoning.

## 2 Materials and methods

### 2.1 Study areas

**Fig. 1:**
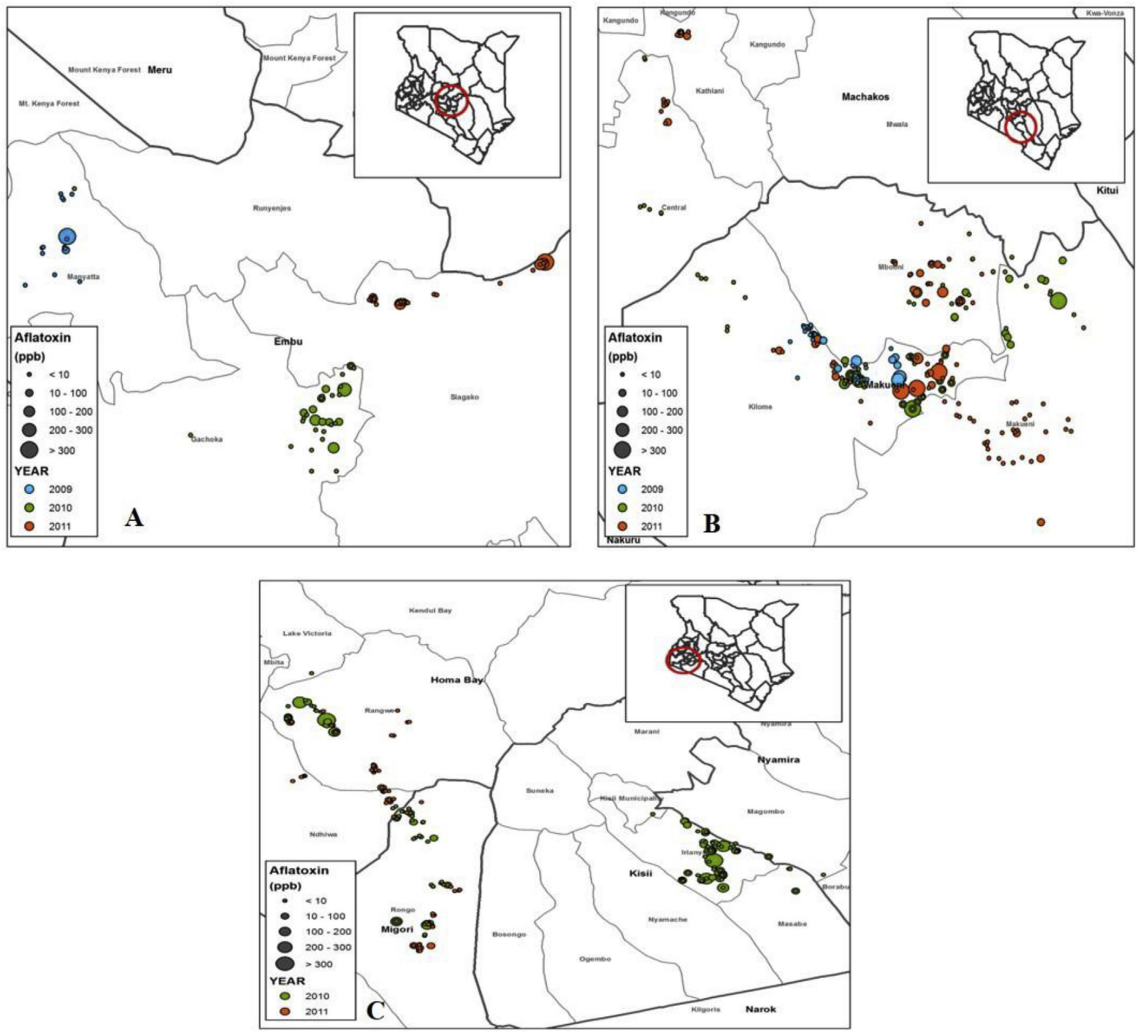
Map showing the distribution of sampling fields and aflatoxin B_1_ levels in samples collected from ready to harvest maize in farmer fields (pre-harvest) in different districts in Eastern and South Western regions of Kenya. (A) location and levels of aflatoxin in samples from Embu, in upper eastern Kenya, (B) samples collected from Machakos and Makueni, lower Eastern Kenya, and (C) samples collected from Kisii, Homa Bay and Migori in South Western region. Samples collected in 2009 are in blue, those collected in 2010 are in green, while those collected in 2011 are magenta in colour. The larger the circle, the higher the levels of aflatoxin in that sample. (For interpretation of the references to color in this figure legend, the reader is referred to the Web version of this article.)

Two regions were selected for this study: Eastern Kenya, a high aflatoxin risk region where acute aflatoxin poisoning has been previously reported (Lewis et al., 2005), and South Western, considered a low risk region with no published reports of aflatoxin poisoning. In Eastern Kenya, samples were collected from three counties: Embu (Fig. 1A), Makueni and Machakos (Fig. 1B), while in South Western, samples were collected from three counties: Homabay, Migori and Kisii (Fig. 1C. Details of the locations within each district where samples were collected are provided in Supplementary Table A1. Both regions have a high maize consumption rate, estimated at 460 g per person per day (Hellin, Chenevix Trench, Kimenju, Narrod, & de Groote, 2011). In the two regions, maize is entirely a rainfed crop and is cultivated in two seasons in a year. In the first season maize is planted between February and April and harvested between July and September, while for the second season it is planted between September and November and harvested between January and April. The timing of planting depends on the onset of rainfall.

### 2.2 Collection of maize samples

Maize samples were collected while the crop was still standing in the field. At the time of collecting samples, the maize was at the R6 stage (physiological maturity). Ten farmers were randomly selected from each village, with sufficient distance (about 5 km) between the villages and farms to obtain a representative sample. Selected fields were approximately 1 acre (0.40 ha) in size, and for each selected field, five quadrants measuring 5 m × 5 m were identified following an initial mapping of the total area constituting the field. Five cobs were randomly selected from each quadrant to give a total of 25 cobs per field. The cobs were hand shelled, and the grain thoroughly mixed to form a composite sample per field. The moisture content of harvested grain was measured, and the grain was dried on plastic mats to 14% moisture content. A one-kilogram sample was taken per farm and transported to the laboratory for aflatoxin extraction and analysis. Samples were stored in a deep freezer (−20 °C) until processing. For each sample, the following information was recorded: farmer's name, village, location and district (GPS coordinates), and name of maize variety.

### 2.3 Sample preparation and aflatoxin analysis

The entire kilogram of maize sample from each field was ground to a fine powder using a dry mill kitchen grinder (Kanchan Multipurpose Kitchen Machine, Kanchan International Limited, Mumbai, India). The milling machine was thoroughly cleaned with compressed air and wiped with paper towels soaked in alcohol and allowed to dry between samples to avoid contamination. Subsequently, a 50 g sub-sample was taken and used to extract aflatoxins following the method described by Mutegi, Ngugi, Hendriks, and Jones (2009). The rest of the sample was packed in 2 kg polythene plastic bags and stored in a deep freezer at −20 °C as a reference sample. For aflatoxin extraction, 50 g maize flour was transferred to a 250-ml flask, and 100 ml of 70% methanol (v/v 70 ml absolute methanol in 30 ml distilled water containing 0.5% w/v potassium chloride) was added. The samples were shaken for 30 min at 300 rpm (Universal Shaker SM 30 C; Edmund Bühler GmbH, Schindäckerstraße 8, Germany). The extract was filtered through Whatman No.41 filter paper and diluted 1:10 in phosphate buffered saline containing 500 μl/l Tween-20 (PBS–Tween). The extracts from the samples were analyzed with an indirect competitive enzyme-linked immunosorbent assay (ELISA) as described by Waliyar, Reddy, and Kumar (2005). Briefly, an aflatoxin–bovine serum albumin conjugate in carbonate coating buffer at 100 ng/ml concentration was prepared and 150 μl dispensed in each well of the Nunc-Maxisorp^®^ ELISA plates (Thermo Fisher Scientific Inc). The plates were incubated at 37 °C for one hour before the toxin solution was collected and stored in a large glass bottle for disposal. The plates were washed in three changes of PBS–Tween, allowing a holding time of 3 min per wash. Next, 200 μl of 0.2% bovine serum albumin (BSA) in PBS–Tween was added to each well and the plates were incubated at 37 °C for one hour. Thereafter, the plates were washed in three changes of PBS–Tween allowing 3 min hold for each wash. AFB_1_ standards (Sigma-Aldrich) at concentrations between 25 and 0.097 ng/ml were prepared in PBST-BSA with 7% methanol; 100 μl per well of AFB1 standards was added into two replicate rows of the ELISA plates. Similarly, 100 μl of each diluted sample extract (1:10 in PBST) was added to two replicate wells. Next, 50 μl of diluted rabbit polyclonal antibody (diluted 1:6000 in PBST-BSA; International Crops Research Institute for the Semi-Arid Tropics, Patancheru, India) was added to all the wells, and the plates were incubated for 1 h at 37 °C. The plates were subsequently washed in three changes of PBS–Tween allowing 3 min hold for each wash. Finally, 150 μl of diluted anti-rabbit–immunoglobulin G–alkaline phosphatase (1:4000 in PBST-BSA) was added to all the wells, and the plates were incubated for 1 h at 37 °C. Thereafter, each well was washed with 150 μl of PBS–Tween. Thereafter, 150 μl of p-Nitrophenyl phosphate, prepared in 10% diethanolamine, pH 9.8, was added to each well, and plates incubated at room temperature. Colour developed in 20–30 min, and the plates were read in a Multiskan Plus reader (Labsystems Company, Helsinki, Finland) at 405 nm. Mean ELISA reading values for each standard and sample were determined. Standard curves were plotted by placing AFB_1_ standard concentration values on the y-axis and optical density values on the x-axis. Regression curves were used to estimate the aflatoxin value in each sample. The lower and upper limits of detection and quantification are 1 and 25 μg/kg AFB_1_, respectively. Samples with aflatoxin concentration >25 μg/kg were diluted with the extraction solvent and re-analyzed. Samples with toxin values below the limit of quantification were considered as containing no detectable toxin. The analytical method used was validated with naturally contaminated corn reference materials (13.4 μg/kg AFB1, product no. TR-A100, batch no. A-C-294; Trilogy Analytical Laboratory, Missouri, USA). In addition, the analytical method was further validated by analyzing randomly selected samples using the VICAM Aflatest (Watertown, MA, USA), as per the manufacturer's instructions.

### 2.4 Estimated exposure to Aflatoxin B_1_

An assessment of daily exposure to toxins in food is dependent on their concentration in the food and the amount consumed. We used three parameters, probable daily intake (PDI), average probable daily intake (APDI), and maximum probable daily intake (MPDI) to assess dietary level exposure to aflatoxins for the population in the study area using the AFB_1_ concentration in the samples. Both PDI and APDI were estimated as described by Herrman and Younes (1999):

1. PDI (ng kg^−1^ body weight (bw) day^−1^) = [maize intake (g person^−1^ day^−1^) × aflatoxin concentration in the maize samples (μg kg^−1^)]/bw (kg);
2. APDI (ng kg^−1^ bw day^−1^) = [maize intake (g person^−1^ day^−1^) × average aflatoxin concentrations in the samples (μg kg^−1^)]/bw (kg).

The estimated maximum probable daily intake (MPDI) of aflatoxin was calculated using the formula:

3. MPDI (ng kg^−1^ bw day^−1^) = (L × D)/bw (kg).

where L is the 90th percentile concentration of aflatoxin in the samples, and D is the daily consumption of maize based foods (g person^−1^ day^−1^). For all estimates, the assumed typical average body weight of an adult was 60 kg, and the average consumption rate for Kenya was 400 g person^−1^ day^−1^ (Muriuki & Siboe, 1995).

### 2.5 Statistical analysis

To characterize the distribution of AFB_1_ in the two regions, samples were grouped into two categories (safe and unsafe) following the legal limits allowed for human consumptions in Kenya (5 μg/kg) (Kenya Bureau of Standards (KEBS) (1988). Samples were considered safe for human consumption if AFB_1_ levels were ≤5 μg kg^−1^ or unsafe if higher. Percentages were computed for frequency of occurrence of each category. AFB_1_ data was log transformed [log_10_(x+1)] before analysis to equalize variances. Data were subjected to analysis of variance (ANOVA) using the general linear model (GLM) procedure of SAS (SAS Institute, 2013). Tukey's Honestly Significant Difference test was used post ANOVA for treatment mean comparison. The *t*-test procedure was used to compare two means. Aflatoxin levels were compared with the maximum safe limits based on East African Community (EAC) (2011) and KEBS (KEBS, 1988) standards.

## 3 Results

### 3.1 Validation of the ELISA assay for aflatoxin quantification

**Fig. 2:**
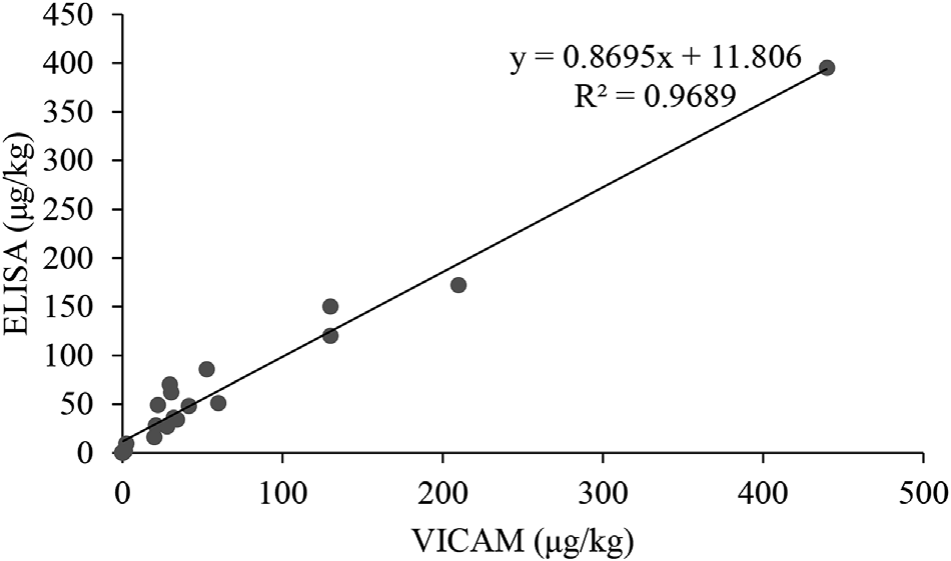
Correlation between aflatoxin B_1_ (AFB_1_) levels obtained using VICAM and ELISA analytical methods. Analysis was done in a subset of 20 randomly selected samples of the maize collected at preharvest.

To validate the utility of ELISA for aflatoxin quantification, 20 samples were randomly selected and assayed using VICAM. ELISA results were highly correlated (r^2^ = 0.98) to results obtained using VICAM (Fig. 2).

### 3.2 Occurrence and levels of Aflatoxin B_1_ in pre-harvest maize samples

**Fig. 3:**
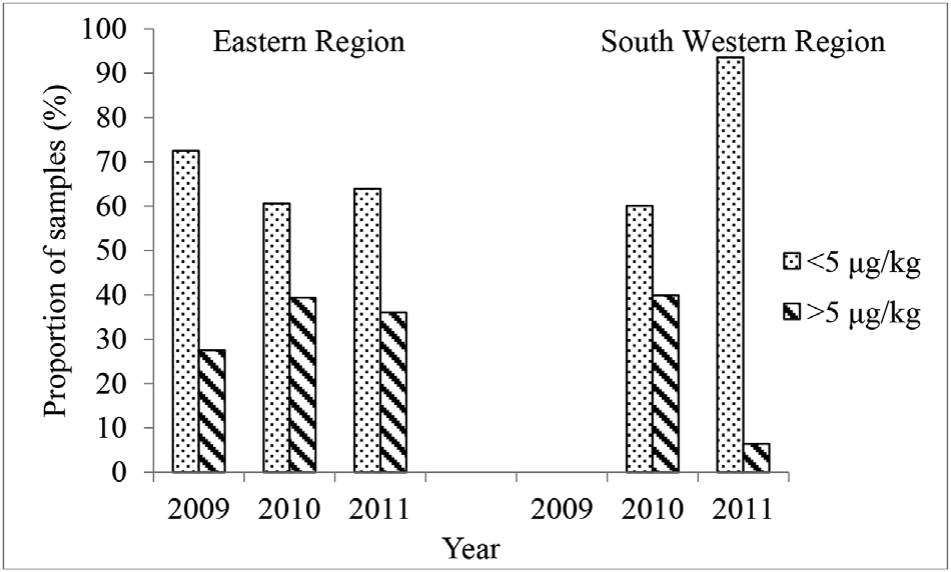
Proportion of maize samples from Eastern and South Western regions with aflatoxin levels below and above allowed limit (5 μg kg^−1^) for maize destined for human consumption. Samples were collected in 2009, 2010 and 2011.

Of the 789 maize samples collected from farmers’ fields in Eastern (416) and South Western (373) regions, AFB_1_ was detected in 274 (65.9%) and 233 (62.5%) samples respectively (Table 1). No statistically significant differences (χ^2^ = 3.316; *P* = 0.069) were observed in the number of samples with detectable AFB_1_ between South Western and Eastern Kenya. A larger proportion of samples from both regions (72.7% for South Western and 63.2% for Eastern) contained either no detectable AFB_1_ or had AFB_1_ levels below 5 μg kg^−1^, the legal limit set by the East African Community (EAC) and the Kenyan authorities as safe for human consumption (Fig. 3). The proportion of samples that had no detectable AFB_1_ was 37.5% for South Western and 34.1% for Eastern region, while the proportion of samples with detectable AFB_1_ but below the 5 μg kg^−1^ threshold level was 35.1% for South Western and 29.1% for Eastern Kenya. Aflatoxin B_1_ levels varied between the regions with significantly higher levels (*P* = 0.0045) in samples from Eastern Kenya (mean, 67.8 μg kg^−1^, median of 6.2 μg kg^−1^) than in South Western (mean, 22.3 μg kg^−1^, median of 4.0 μg kg-1). The most highly contaminated sample in Eastern Kenya contained 9091. 8 μg kg^−1^ AFB_1_, whereas in South Western, the highly contaminated sample contained 722.2 μg kg^−1^. Although the proportion of samples with aflatoxin levels above the maximum tolerable limit of 5 μg kg^−1^ were higher for Eastern Kenya (55.8%) compared to South Western (43.8%), the difference was not statistically significant (*P* > 0.05). Eastern Kenya had a significantly higher number of samples with AFB1 levels in the 10–100 μg kg^−1^ range (81) compared to South Western region (50), and the highest levels of contamination were obtained in Eastern Kenya (Fig. 1).

**Table 1:** Prevalence and descriptive statistics of aflatoxin B_1_ (AFB_1_) in maize collected from farmers’ fields in eastern and South Western regions of Kenya and proportion of maize samples with AFB_1_ levels exceeding maximum tolerable limits in the East African Community (EAC) from 2009 to 2011.

Region	Year	N	Positive samples		Aflatoxin concentration range of positive samples (μg kg^−1^)	Median (μg kg^−1^)	Mean ± SD (μg kg^−1^)	Proportion of positive samples exceeding EAC regulatory limit (≥5 μg kg^−1^)	
			N	%				N	%
Eastern	2009	40	13	32.5	2.0–9091.8	45.7	775.5 ± 2500.2	11	84.6
	2010	193	127	65.8	0.1–1454.8	7.4	39.1 ± 147.5	76	59.8
	2011	183	134	72.2	1.0–581.5	4.9	26.3 ± 71.4	66	49.3
	**Total**	**416**	**274**	**65.9**	**0.01**–**9091.8**	**6.2**	**67.8 ± 558.9**	**153**	**55.8**

South Western	2010	233	157	67.4	1.0–722.2	6.9	31.0 ± 83.5	93	59.2
	2011	140	76	54.3	0.9–63.1	2.2	4.1 ± 7.7	9	11.8
	**Total**	**373**	**233**	**62.5**	**0.98–722.2**	**4.01**	**22.3 ± 69.8**	**102**	**43.8**
Overall		**789**	**507**	**64.3**	**0.01–9091.8**	**5.2**	**46.9 ± 413.9**	**255**	**50.3**

### 3.3 Levels of AFB_1_ contamination between years

There were no significant differences (*P* = 0.9900) in levels of AFB_1_ between South Western and Eastern Kenya in 2010, whereas significantly higher levels of AFB_1_ (*P* < 0.0001) were observed in Eastern Kenya (average AFB_1_ = 26.3 μg kg^−1^) than in South Western (average AFB_1_ = 4.1 μg kg^−1^) in 2011 (Table 1). Within a region, no statistically significant differences (*P* = 0.05) were observed in the number of samples with aflatoxin levels above 5 μg kg^−1^ for 2009 (84.6%), 2010 (59.8%) and 2011 (49.3%) for Eastern Kenya. However, average AFB_1_ levels were higher for 2009 (775.5 μg kg^−1^) and this was significantly different (*P* < 0.001) from average aflatoxin levels for 2010 and 2011. In South Western, highly significant differences (*P* < 0.001) were observed between years for both the proportion of samples above the legal limit of 5 μg kg^−1^ and the average levels of AFB_1_.

### 3.4 AFB_1_ levels in different districts within a region

Significantly higher levels of aflatoxins were observed in Embu (mean = 196.3 μg kg^−1^) followed by Makueni (mean = 39.0 μg kg^−1^) in Easter region (Table 2). Although statistically not significant, the proportion of samples with AFB_1_ levels greater than 5 μg kg^−1^ followed a similar trend as the aflatoxin level. In South Western, Kisii had the highest AFB1 levels (mean = 28.5 μg kg^−1^) followed by Homabay (mean = 24.5 μg kg^−1^). The proportion of samples with AFB_1_ levels above 5 μg kg^−1^ were highest in Kisii (54.4%) followed by Migori (42.7%).

**Table 2:** Prevalence and descriptive statistics of aflatoxin B_1_ (AFB_1_) in maize collected from farmers’ fields in different districts of Eastern and South Western Kenya and proportion of maize samples with AFB_1_ levels exceeding maximum tolerable limits set by the East African Community (EAC) and Kenyan authorities.

Region	District	N	Positive samples		Aflatoxin concentration range of positive samples (μg kg^−1^)	Median (μg kg^−1^)	Mean ± SD (μg kg^−1^)	Proportion of positive samples exceeding EAC and KEBS regulatory limit (≥5 μg kg^−1^)	
			N	%				N	%
Eastern	Embu	89	57	64.0	0.95–9091.8	6.25	196.3 ± 1202.5	34	59.6
	Machakos	62	38	61.3	1.3–70.93	3.69	10.5 ± 16.5	15	39.5
	Makueni	265	179	67.5	0.01–1454.8	6.90	39.0 ± 131.5	104	58.1

South Western	Homa Bay	122	68	55.7	0.98–722.2	2.59	24.5 ± 94.9	21	30.9
	Kisii	118	90	76.3	1.0–558.7	6.30	28.5 ± 72.7	49	54.4
	Migori	133	75	56.4	0.98–120.7	3.81	12.7 ± 24.9	32	42.7

### 3.5 Exposure to aflatoxins

Based on AFB_1_ levels in the analyzed maize samples, the probable daily intake (PDI) of AFB_1_ was estimated to range from 0.07 to 60612 ng kg^−1^ bw day^−1^ in Eastern and from 6.53 to 4814.70 ng kg^−1^ bw day^−1^ in South Western Kenya (Table 3). Average population exposure to AFB_1_ was significantly higher in Eastern (451.8 ng kg^−1^ bw day^-^1) than in South Western Kenya (148.4 ng kg^−1^ bw day^-^1). The average PDI was highest in Embu district for the Eastern region, and Kisii in South Western region. Considering the high levels of maize consumption based on 90^th^ percentile, the exposure measured as MPDI was 282.9 ng kg^−1^ bw day^−1^ in South Western and 572.9 ng kg^−1^ bw day^−1^ in Eastern Kenya. The overall APDI of AFB_1_ across the two regions was 312.4 ng kg^−1^ bw day^−1^, while for excessive consumers, the MPDI was 362 ng kg^−1^ bw day^−1^ (Table 3; Supplemental Table A2).

**Table 3:** Aflatoxin B_1_ concentration, probable daily intake (PDI), average probable daily intake (APDI) and maximum probable daily intake (MPDI) of AFB_1_ in maize consumed by region and districts of Kenya.

Region	District	Aflatoxin concentration range (μg kg^−1^)	Median concentration (μg kg^−1^)	90^th^ percentile concentration (μg kg^−1^)	PDI range ng kg^−1^ bw day^−1^^a^	APDI ng kg^−1^ bw day^−1^^a^	MPDI ng kg^−1^ bw day^−1^ (90^th^ percentile)^a^
Eastern	Embu	0.95–9091.8	6.25	125.11	6.33–61	1308.56	834.07
	Machakos	1.30–70.93	3.69	39.03	8.67–472.87	69.81	260.20
	Makueni	0.01–1454.79	6.90	85.93	0.07–9698.60	260.02	572.87
		0.01–9091.8	6.23	85.93	0.07–60612.00	451.80	572.90

South Western	Homa Bay	0.98–722.2	2.59	51.30	6.53–4814.67	163.45	342.00
	Kisii	1.00–558.7	6.30	54.93	6.67–3724.67	190.07	366.20
	Migori	0.98–120.7	3.81	34.90	6.53–804.67	84.86	232.67
		0.98–722.2	4.00	42.40	6.53–4814.70	148.40	282.9
	**Overall**			**54.30**	**0.07–60612.00**	**312.4**	**362.00**

a Estimated average body weight was: 60 kg, while average per capita consumption rate was 400 g/day per person day^−1^.

### 3.6 Maize varieties grown in the sampled districts

In all areas sampled, farmers grew both hybrids and open pollinated local varieties (Supplemental Table A3). In both South Western and Eastern, local varieties were favored by farmers, accounting for 36% of all samples collected. Several hybrids (11) were common between the two regions. Aflatoxin B_1_ was detected in all varieties and hybrids sampled and no significant differences were observed in levels of AFB_1_ (Supplemental Fig. 1), revealing that these varieties were equally vulnerable to aflatoxin contamination. Of the varieties that were common between Eastern and South Western, no significant differences were observed (*P* > 0.05) in AFB_1_ levels between the two regions (data not shown).

## 4 Discussion

Kenya has a history of acute aflatoxicosis, stemming from consumption of maize with high levels of aflatoxin, with cases reported only in Eastern Kenya (Ngindu et al., 1982; Lewis et al., 2005; Daniel et al., 2011; Yard et al., 2013; Kang’ethe et al., 2017). Most studies to document incidence of aflatoxicosis have been conducted in the high-risk region of Kenya, and usually following cases of acute aflatoxicosis. This study was carried out to document the incidence and prevalence of aflatoxin contamination of physiologically mature maize in the field from a region of Kenya with high maize consumption but no reported cases of aflatoxin poisoning and compare results to a region with reported high incidences of aflatoxin poisoning. Results from this study showed that maize from farmers' fields in the low aflatoxin risk region of South Western was equally contaminated with aflatoxin as maize from the high risk Eastern region. However, AFB_1_ levels were higher in eastern Kenya; concurring with reports by Yard et al. (2013), who reported that aflatoxin exposure varied with region and highest levels were detected in the Eastern, compared to other regions. The cause of the high levels of aflatoxins in Eastern Kenya is not known, but it could be related to prevailing climatic conditions as well as presence of different strains of *A. flavus* (Probst, Bandyopadhyay, & Cotty, 2014). High incidences of drought and high temperatures have been reported in Eastern Kenya (Mutunga, Ndungu, & Muendo, 2017; PDNA, 2012) and these parameters have been shown to exert positive impact on establishment and proliferation of *A. flavus* and subsequent aflatoxin contamination (Widstrom, McMillian, Beaver, & Wilson, 1990). Furthermore, Eastern Kenya has been reported to have high incidences of S-morphotype of *A. flavus*, a strain known to produce high levels of aflatoxin (Probst et al., 2014). Further research is needed to characterize *A. flavus* strains in South Western region to better understand the differences in incidences of aflatoxin levels compared in Eastern Kenya.

Numerous studies mapping the incidence of aflatoxin poisoning focused on regions with acute aflatoxicosis (Kilonzo, Imungi, Muiru, Lamuka, & Njage, 2014; Lewis et al., 2005; Yard et al., 2013), and no study has covered other regions with no reported cases of acute aflatoxicosis have not been reported, and in preharvest maize. Our study is the first to investigate aflatoxin contamination in multiple years without acute aflatoxicosis and assessing both high and low risk areas to understand the extent of contamination in pre-harvest maize. In this study, 50.3% samples had AFB_1_ levels exceeding the legal limit (5 μg kg^−1^) and the highly contaminated sample had 9091.8 μg kg^−1^ and was found in the Eastern region. The AFB_1_ concentration recorded in this study is much higher than that reported in preharvest maize in Benin (2–2500 μg kg^−1^) by Sétamou, Cardwell, Schulthess, and Hell (1997). Variable levels of aflatoxin contamination ranging from 0 to 2760 ng g^−1^ (Zuber, Calvert, Lillehoj, & Kwolek, 1976) and from 1 to 4708 μg kg^−1^ (McMillian, Wilson, & Widstrom, 1985) in preharvest maize have been reported in temperate maize. High aflatoxin levels, up to 48,000 μg kg^−1^ have been reported in maize collected from farmer storage structures or sourced from markets in Kenya (Daniel et al., 2011). Lower levels have also been reported in maize at harvest in South Western by Mutiga, Hoffmann, Harvey, Milgroom, and Nelson (2015), the same region targeted in this study. This study revealed that high levels of AFB_1_ can be found in maize that is still standing in the field just after physiological maturity.

Levels of aflatoxin contamination varied from year to year and from one sampling point to the other. This probably reflects the non-homogenous distribution of aflatoxin within maize lots and prevailing environmental conditions (Hamidou, Rathore, Waliyar, & Vadez, 2014; Widstrom et al., 1990). Contamination of maize by *A. flavus* and aflatoxin has been reported to be high under conditions that stress the crop, such as high temperatures, drought and damage from other abiotic and biotic factors (Hamidou et al., 2014; Hawkins, Windham, & Williams, 2005). Maize in Kenya is rain-fed and the crop is vulnerable to large variability in environmental conditions, especially frequent droughts that have been recorded. For example, a severe drought was experienced in 2009 that led to failure of the crop in most of Eastern Kenya (PDNA, 2012) and Western Kenya (Reynolds, 2009). Similarly, higher levels of aflatoxin contamination were recorded in 2009, suggesting a correlation between drought and aflatoxin contamination. We did not assess insect infestation in the maize samples collected but insects are known to influence mycotoxin contamination (Sétamou et al., 1997).

Proper storage and grain moisture management have been suggested as critical aflatoxin contamination control interventions (Walker, Jaime, Kagot, & Probst, 2018). As such, most aflatoxin intervention programs have been focusing on post-harvest management. However, our study revealed that maize is already contaminated before it reaches the store; thus, little can be done to prevent contamination. Good post-harvest handling and storage will prevent further accumulation of aflatoxins but will do nothing to reduce aflatoxin levels already present in maize before harvest. The presence of a large number of pre-harvest maize samples contaminated by aflatoxin, in both South Western and Eastern regions reveals the importance of developing strategies targeted at minimizing aflatoxin contamination while the crop is still in the field. This is a critical step in minimizing aflatoxin contamination. Technologies, such as the use of resistant maize varieties, good crop husbandry to minimize damage from insects and diseases, and proper fertilization schemes can go a long way to minimize infection by *A. flavus* and subsequent contamination with aflatoxin. Adopting technologies that minimize stress by combining heat and drought tolerant maize lines with high levels of resistance to *A. flavus* and aflatoxin contamination should be emphasized (Cairns et al., 2013; Mahuku, Warburton, Makumbi, & San Vicente, 2013). Biological control using atoxigenic strains of *A. flavus* to prevent infection by toxigenic strains has been found to consistently reduce aflatoxin contamination by >80% (Bandyopadhyay et al., 2016) and should be promoted to become an integral part of pre-harvest aflatoxin control measures.

Maize is the major staple for Kenya, with an average consumption rate of 400 g day^−1^ person^−1^ (Hellin et al., 2011; Muriuki & Siboe, 1995). Cases of acute aflatoxin poisoning have occurred among people living in rural, subsistence farming communities and consuming homegrown maize (Azziz-Baumgartner et al., 2005; Kilonzo et al., 2014). All farmers who participated in the current study produced maize for own consumption and sold the surplus in local markets. This study revealed that populations in the study districts in South Western are equally exposed and vulnerable to AFB_1_ health hazards as the population in Eastern Kenya. Exposure to AFB_1_ was high in both South Western (148.4 ng kg^−1^ bw day^−1^) and Eastern (451.8 ng kg^−1^ bw day^−1^) regions of Kenya. This result suggests that the population is continuously in danger of suffering from the effects of aflatoxins. The values of PDI recorded in nearly all the districts covered in this study were higher than those reported by Adetunji et al. (2014). Our results of PDI are higher than reported by Mendoza et al. (2018) (0.01–0.85 ng kg^−1^ bw day^−1^) for Gatemala, Park et al. (2004) (1.19–5.79 ng kg^−1^ bw day^−1^) for Korea, and Jager, Tedesco, Souto, and Oliveira (2013) for Brazil. Considering the reports of the Joint FAO/WHO Expert Committee on Food Additives (JECFA) (JECFA, 1999) and Scientific Committee on Food (SCF) (SCF, 1994), that a very low exposure level to aflatoxins (1 ng kg^−1^ bw/day) may induce liver cancer cases, the average and median exposure rates observed in this study reveal a high risk of aflatoxin poisoning for the population in the two regions. As several reports indicate (Azziz-Baumgartner et al., 2005; Lewis et al., 2005; Ngindu et al., 1982), cases of acute aflatoxicosis have only been reported from Eastern Kenya and none in South Western region. Socio-economic studies and detailed nutrition/diet analyses are needed to understand why no cases of aflatoxicosis have been reported in South Western region, even though the levels of possible aflatoxin exposure are high and comparable to Eastern Kenya. This information might inform strategies to recommend for minimizing aflatoxin poisoning in Eastern Kenya.

In this study, we observed that different maize varieties and hybrids are grown by farmers, and in some cases, farmers grew both improved and local varieties as security against crop failure from adverse climatic conditions that are frequently encountered during the maize growing seasons. No significant differences were found in the levels of aflatoxin between the different varieties, revealing that the currently grown maize germplasm is not adequate for managing *A. flavus* infection and subsequent contamination by aflatoxin. Resistance to *A. flavus* and aflatoxin accumulation has been reported in tropical maize inbred lines (Brown et al., 2013) but commercially grown hybrids have not been released yet. More efforts are required to develop maize hybrids and varieties that are tolerant or resistant to infection by *A. flavus* and subsequent contamination by aflatoxins. Furthermore, stress from abiotic (drought, heat, and poor soil fertility) and biotic factors have been shown to increase *A. flavus* infection and aflatoxin contamination (Hamidou et al., 2014; Hawkins et al., 2005). Good sources of drought and heat tolerance have been developed by CIMMYT (Cairns et al., 2013). Therefore, new research efforts should focus on combining drought and heat tolerance with *A. flavus* resistance to develop high yielding stable hybrids and open-pollinated varieties suitable for the diverse agroecologies in Kenya and overall in sub-Saharan Africa.

## 5 Conclusion

This study showed that infection of maize by *A. flavus* and subsequent aflatoxin contamination starts under field conditions and this is the critical step to focus aflatoxin prevention strategies. Developing strategies targeted at minimizing aflatoxin contamination while the crop is still in the field should be given high priority. This study documented exposure in two regions in Kenya, including a region with no prior reports of exposure and no documented aflatoxicosis outbreaks. Our results demonstrate an urgent need to implement evidence-based interventions in Kenya to decrease aflatoxin exposure and subsequently avert adverse health effects. More studies are required to better understand the levels of aflatoxin contamination and burden across Kenya.

## Conflicts of interest

The authors declare that there are no conflicts of interest.

## Funding

This work was supported by the Bill and Melinda Gates Foundation, Seattle, WA through Aflacontrol – Aflatoxin risk reduction strategies project (grant number 51892).

## Abbreviations

AFB_1_: aflatoxin B_1_
ELISA: enzyme-linked immunosorbent assay
EAC: East African Community
KEBS: Kenya Bureau of Standards
PDI: probable daily intake
APDI: average probable daily intake
MPDI: maximum probable daily intake
