# Supplementary material for: Pre-harvest management is a critical practice for minimizing aflatoxin contamination of maize
Source: Food Control. 2019 Feb;96:219–26. doi: 10.1016/j.foodcont.2018.08.032 (PMC6251936; doi:10.1016/j.foodcont.2018.08.032)
Supplement: Multimedia component 1 [file mmc1.docx]

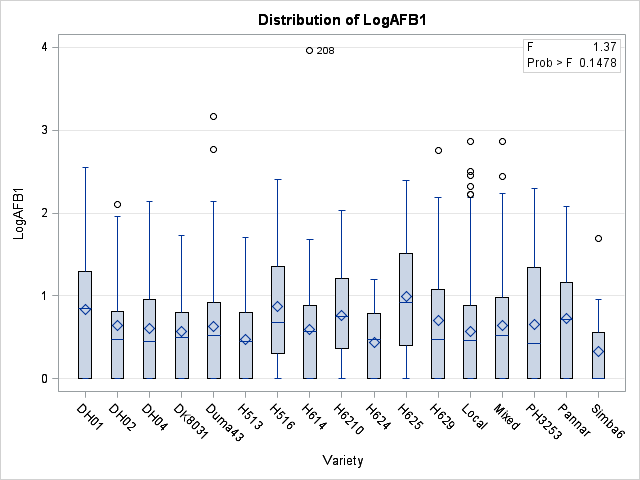


**Supplementary Figure 1**: Aflatoxin B_1_ levels (logAFB_1_) in maize hybrids and varieties grown in Eastern and South Western regions of Kenya.
