# Supplementary material for: Pre-harvest management is a critical practice for minimizing aflatoxin contamination of maize
Source: Food Control. 2019 Feb;96:219–26. doi: 10.1016/j.foodcont.2018.08.032 (PMC6251936; doi:10.1016/j.foodcont.2018.08.032)
Supplement: Multimedia component 2 [file mmc2.docx]

**Supplementary Table A.1.** Region, district, location, and number of villages and samples collected from the high (Eastern) and low aflatoxin (South Western) risk regions in Kenya.

| Region | District | Division | Location | Number of villages sampled | Number of samples collected |
| --- | --- | --- | --- | --- | --- |
| Eastern | Embu | Evurore | Kanyuambora | 3 | 31 |
|  |  |  | Ishiara | 2 | 9 |
|  |  | Manyatta | Runguru | 8 | 11 |
|  |  | Siakago | Nthawa | 10 | 38 |
|  | **Total** |  |  | **23** | **89** |
|  |  |  |  |  |  |
|  | Machakos | Kathiani | Kanzalu | 1 | 1 |
|  |  |  | Mitaboni | 2 | 10 |
|  |  | Kyanzavi | Matuu | 2 | 2 |
|  |  | Machakos | Kivutini | 1 | 5 |
|  |  |  | Mumbuni | 1 | 5 |
|  |  | Matungulu | Kalandini | 2 | 7 |
|  |  |  | Tala | 1 | 3 |
|  |  | Central | Kaheka-kai | 2 | 7 |
|  |  |  | Ngelani | 1 | 3 |
|  |  | Kalama | Kola | 1 | 3 |
|  |  |  | Lumbwa | 1 | 4 |
|  |  | Kangundo | Isinga | 1 | 1 |
|  |  |  | Kangundo | 3 | 11 |
|  | **Total** |  |  | **19** | **62** |
|  |  |  |  |  |  |
|  | Makueni | Kaiti | Ukia | 21 | 82 |
|  |  |  | Luani | 5 | 11 |
|  |  |  | Kilala | 11 | 31 |
|  |  |  | Kiniani | 5 | 31 |
|  |  |  | Nthangu | 2 | 2 |
|  |  | Kalawa | Kalawa | 10 | 12 |
|  |  | Kisau | Kako | 2 | 6 |
|  |  |  | Kisau | 13 | 32 |
|  |  |  | Kiteta | 1 | 1 |
|  |  |  | Ngoni | 1 | 4 |
|  |  |  | Mang'ani | 1 | 1 |
|  |  | Wawa | Sakai | 4 | 8 |
|  |  |  | Waia | 5 | 11 |
|  |  | Wote | Kikumini | 11 | 16 |
|  |  |  | Kilala | 1 | 1 |
|  |  |  | Muvau | 1 | 3 |
|  |  |  | Wote | 6 | 13 |
|  | **Total** |  |  | **100** | **265** |
| South Western | Homa Bay | Asego | Kanyada | 5 | 25 |
|  |  |  | Kanyada East | 3 | 16 |
|  |  |  | Kanyada West | 4 | 17 |
|  |  |  | Kanyada North | 4 | 10 |
|  |  |  | GEM East | 6 | 13 |
|  |  |  | Homa Bay | 2 | 2 |
|  |  | Ndhiwa | Kanyamua | 1 | 3 |
|  |  |  | North Kanyamua | 4 | 13 |
|  |  | Riana | East Kaboch | 3 | 10 |
|  |  |  | Kaboch | 3 | 13 |
|  | **Total** |  |  | **35** | **122** |
|  |  |  |  |  |  |
|  | Kisii | Keumbu | Ibeno | 5 | 23 |
|  |  |  | Iterio | 1 | 3 |
|  |  |  | Kegati | 3 | 17 |
|  |  |  | Keumbu | 10 | 62 |
|  |  | Kiamokama | Ekemuga | 1 | 3 |
|  |  | Kissi | Keumbu | 1 | 3 |
|  |  | Rigoma | East Kitutu | 2 | 7 |
|  | **Total** |  |  | **23** | **118** |
|  |  |  |  |  |  |
|  | Migori | Awendo | Sakwa | 2 | 7 |
|  |  | Rongo | East Kamagambo | 11 | 51 |
|  |  |  | Kamagambo | 7 | 24 |
|  |  |  | South Kamagambo | 2 | 8 |
|  |  |  | Sakwa | 6 | 24 |
|  |  |  | Sakwa East | 2 | 14 |
|  |  |  | Sakwa North East | 1 | 5 |
|  | **Total** |  |  | **31** | **133** |
|  | **Total number of samples** |  |  | **231** | **789** |

**Supplementary Table A.2**. Concentration, probable daily intake (PDI), average probable daily intake (APDI) and maximum probable daily intake (MPDI) of aflatoxins in pre-harvest maize in different districts of Kenya.

|  |  |  |  |  | Aflatoxin levels in positive samples (µg kg^-1^) | | | |  |  |  |
| --- | --- | --- | --- | --- | --- | --- | --- | --- | --- | --- | --- |
| Region | Year | District | N | Positive N (%) | Concentration range | Mean ± STD | Median | 90^th^ percentile concentration | PDI range ng kg^-1^ bw day^-1^ | APDI ng kg^-1^ bw day^-1^ | MPDI ng kg^-1^ bw day^-1^ (90th percentile) |
| Eastern | 2009 | Makueni | 30 | 11 (36.7%) | 2.0 - 273.8 | 89.7 ± 89.8 | 45.7 | 200.8 | 13.33 - 1825.33 | 598.00 | 1338. 67 |
|  | 2009 | Embu | 10 | 2 (20%) | 2.8 -9091.8 | 4547.3 ± 6426.9 | 4547.3 | 9091.8 | 18.67 -60612 | 30315.33 | 60612 |
|  |  |  |  |  |  |  |  |  |  |  |  |
|  | 2010 | Machakos | 22 | 13 (59.1%) | 2.2 - 9.3 | 4 ± 2.5 | 3.3 | 8.6 | 14.67 -62.00 | 26.67 | 57.33 |
|  | 2010 | Makueni | 132 | 89 (67.4%) | 0.01 – 1454.8 | 44.1 ± 172.8 | 8.0 | 60.2 | 0.067 - 9698.67 | 294 | 401.33 |
|  | 2010 | Embu | 39 | 25 (64.1%) | 0.95 – 251.98 | 39.4 ± 63.6 | 8.6 | 154.2 | 6.67 -1680 | 262. 67 | 1028 |
|  |  |  |  |  |  |  |  |  |  |  |  |
|  | 2011 | Machakos | 40 | 25 (62.5) | 1.3 - 70.9 | 13.9 ± 19.6 | 4.7 | 48.14 | 8.67 - 472.67 | 92. 67 | 353.33 |
|  | 2011 | Makueni | 103 | 79 (76.7) | 0.99 - 354.6 | 26.16 ± 64.5 | 5.17 | 48.23 | 6.6 - 2364 | 174.4 | 321.53 |
|  | 2011 | Embu | 40 | 30 (75%) | 1.1 -581.5 | 37 ± 107.9 | 4.8 | 103.3 | 7.33 - 3876.67 | 246. 67 | 688. 67 |
|  |  |  |  |  |  |  |  |  |  |  |  |
|  |  |  |  |  |  |  |  |  |  |  |  |
| South Western | 2010 | Homabay | 61 | 35 (57.4%) | 0.99 - 722.2 | 44.5 ± 129.9 | 4.7 | 74.9 | 6.67 - 4814.67 | 296. 67 | 499.33 |
|  | 2010 | Kisii | 78 | 68 (87.2) | 1.0 - 558.7 | 36 ± 82.1 | 8.7 | 119.9 | 6.67 - 3724.67 | 240.00 | 799.33 |
|  | 2010 | Migori | 94 | 54 (57.4%) | 1.3 - 120.7 | 16.1 ± 28.6 | 5.3 | 40.9 | 8.67 - 804.67 | 107.33 | 272. 67 |
|  |  |  |  |  |  |  |  |  |  |  |  |
|  | 2011 | Homa Bay | 61 | 33 (54.1) | 0.98 - 20.2 | 3.3 ± 3.9 | 2.1 | 5.3 | 6.67 - 134.67 | 22.00 | 35.33 |
|  | 2011 | Kisii | 40 | 22 (55%) | 1.1 - 63.1 | 5.3 ± 12.96 | 2.5 | 4.68 | 7.33 - 420.67 | 35.33 | 31.20 |
|  | 2011 | Migori | 39 | 21 (53.8%) | 0.98 - 16.6 | 4.2 ± 4.63 | 2.5 | 13.6 | 6.67 - 110.67 | 28.00 | 90. 67 |

**Supplemental Table A.3**: Maize varieties, hybrids or landraces grown by farmers in eastern and South Western regions in Kenya, frequency of occurrence and average aflatoxin levels.

| **Maize variety / hybrid** | **Frequency of occurrence** | **Mean AFB_1_ level (µg kg^-1^)** | **Region of Kenya** |
| --- | --- | --- | --- |
| DH01 | 3.06 | 36.01 | Eastern |
| DH02 | 2.68 | 15.53 | Eastern |
| DH04 | 2.93 | 14.38 | Eastern |
| DK8031 | 3.82 | 28.97 | Eastern/South Western |
| Duma43 | 8.41 | 20.76 | Eastern/South Western |
| H505 | 0.64 | 2.74 | South Western |
| H513 | 1.66 | 6.92 | Eastern/South Western |
| H5I6 | 1.91 | 56.34 | Eastern/South Western |
| H614 | 6.11 | 3.88 | Eastern/South Western |
| H6210 | 2.68 | 8.97 | South Western |
| H6213 | 0.76 | 24.24 | South Western |
| H624 | 2.17 | 3.00 | South Western |
| H625 | 1.91 | 40.48 | South Western |
| H628 | 0.38 | 0.99 | South Western |
| H629 | 1.53 | 50.84 | South Western |
| H691 | 0.64 | 63.43 | South Western |
| H9401 | 0.51 | 1.04 | South Western |
| KCB | 1.40 | 10.44 | Eastern |
| Local | 36.31 | 14.61 | Eastern/South Western |
| MixedH | 1.27 | 23.03 | Eastern/South Western |
| MixedL | 7.26 | 24.20 | Eastern/South Western |
| Pannar | 2.29 | 26.97 | Eastern/South Western |
| PH04 | 1.15 | 7.50 | Eastern/South Western |
| PH3253 | 5.35 | 11.43 | Eastern/South Western |
| Simba61 | 3.18 | 1.74 | South Western |
